# Perioperative Supplemental Oxygen for the Prevention of Surgical Site Infection After Cesarean Section: A Systematic Review of Existing Literature

**DOI:** 10.7759/cureus.65244

**Published:** 2024-07-24

**Authors:** Avir Sarkar, Neelima Choudhary, Sivaranjani P, Shivam Pandey, Ghanashyam Bhoi, Charu Taneja

**Affiliations:** 1 Obstetrics and Gynecology, Noida Institute of Medical Sciences, Greater Noida, IND; 2 Obstetrics and Gynecology, Employees' State Insurance Corporation (ESIC) Medical College and Hospital Faridabad, Faridabad, IND; 3 Obstetrics and Gynecology, All India Institute of Medical Sciences, Kalyani, Kalyani, IND; 4 Biostatistics, All India Institute of Medical Sciences, New Delhi, New Delhi, IND

**Keywords:** ceserean delivery, high oxygen, surgical site infection, supplemental oxygen, fio2

## Abstract

Initial systematic reviews demonstrated the reduction of surgical site infection (SSI) following perioperative oxygen supplementation. SSI among colorectal surgeries was reduced by more than 50% with high-flow oxygen. However, recent randomized trials are coming up with conflicting results.

The objective of this review was to comprehend whether the application of perioperative supplemental oxygen decreased the hazard of SSI following cesarean delivery. The initial search identified 95 studies. After screening title and abstracts 59 studies were included, and 33 studies were found to be relevant after checking eligibility. After a careful analysis, five articles were found fit for this systematic review. Extracted information included study design and methodology, the cumulative incidence of post-cesarean SSI following supplemental oxygen, the odds ratio, and associated variability for all factors considered in univariate and/or multivariate analysis.

The cumulative incidence of the standard care group and supplemental oxygen group were comparable in all five studies with statistically significant differences. The secondary outcomes such as hospital readmission, wound separation, and intravenous antibiotics were similar in both groups as stated in the two studies. The rate of SSI in diabetics was 6.9% and 14.4% in the standard care group and supplemental oxygen group, respectively, as analyzed in a study. An increase in intra-operative blood loss was found to be the major risk factor leading to SSI. In one of the studies, Caucasian race, increased basal metabolic index, and prolonged surgery were associated with increased risk of SSI. There was no difference in neonatal umbilical artery pH resulting from supplemental oxygen during cesarean. The available literature is quite sufficient to prove that supplemental oxygen offers no added benefit in reducing post-cesarean SSI. Hence, we do not recommend its use for this purpose.

## Introduction and background

Although initial studies from both human and animal models have demonstrated a decreased incidence of surgical site infection (SSI) following increased tissue oxygen tension by administration of a high fraction of inspired oxygen (FiO_2_) [1-3], recent randomized trials are coming up with conflicting results. The prevalence of SSI after cesarean section ranges from 3% to 15% across countries [1,2]. The landmark trial by Grief et al. showed a decrease in the risk of SSI by more than 50% during colorectal surgeries after administration of 80% FiO_2_ [3]. However, further trials debated this concept [4,5]. Some suggest high FiO_2_ to be an economical, minimal, and safe intervention, while others indicated it to have no impact on the SSI frequency.

Regardless of the contradictory articles, the World Health Organization (WHO) Guidelines Development Group in 2016 recommended that adults requiring general anesthesia during surgery should have an 80% FiO_2_ intraoperatively, and if possible, even up to six hours subsequently so as to decrease the hazard of SSI (level I recommendation, moderate quality of evidence) [6]. The main objective of this review was to comprehend whether the application of this recommendation was helpful in reducing SSI following cesarean section and whether this strength of recommendation needs reconsideration. Hence, we have summarized the trials acknowledging the link between supplemental oxygen and SSI following cesarean section in order to stipulate a thorough quantitative deduction concerning this subject. In this review, we aimed to assess whether supplemental high-flow oxygen (FiO_2_ - 80%) during the perioperative period was effective in reducing the incidence of SSI following cesarean section.

## Review

Systematic literature search

Preferred Reporting Items for Systematic Reviews and Meta-Analyses (PRISMA) 2020 guideline was used in this systematic review [7]. A literature search was done and all published text pertaining to the effect of supplemental oxygen in reducing the incidence of SSI after cesarean deliveries. The result of this systematic review would help guide us to modify our perioperative anesthetic measures during high-risk cesarean sections in the near future. During the process of this review, the following steps were conducted: a) initially, the research question was determined, b) following which an extensive literature search was conducted, c) after which, all appropriate studies were recruited, d) and finally, the data was presented in a tabular format.

**Figure 1:**
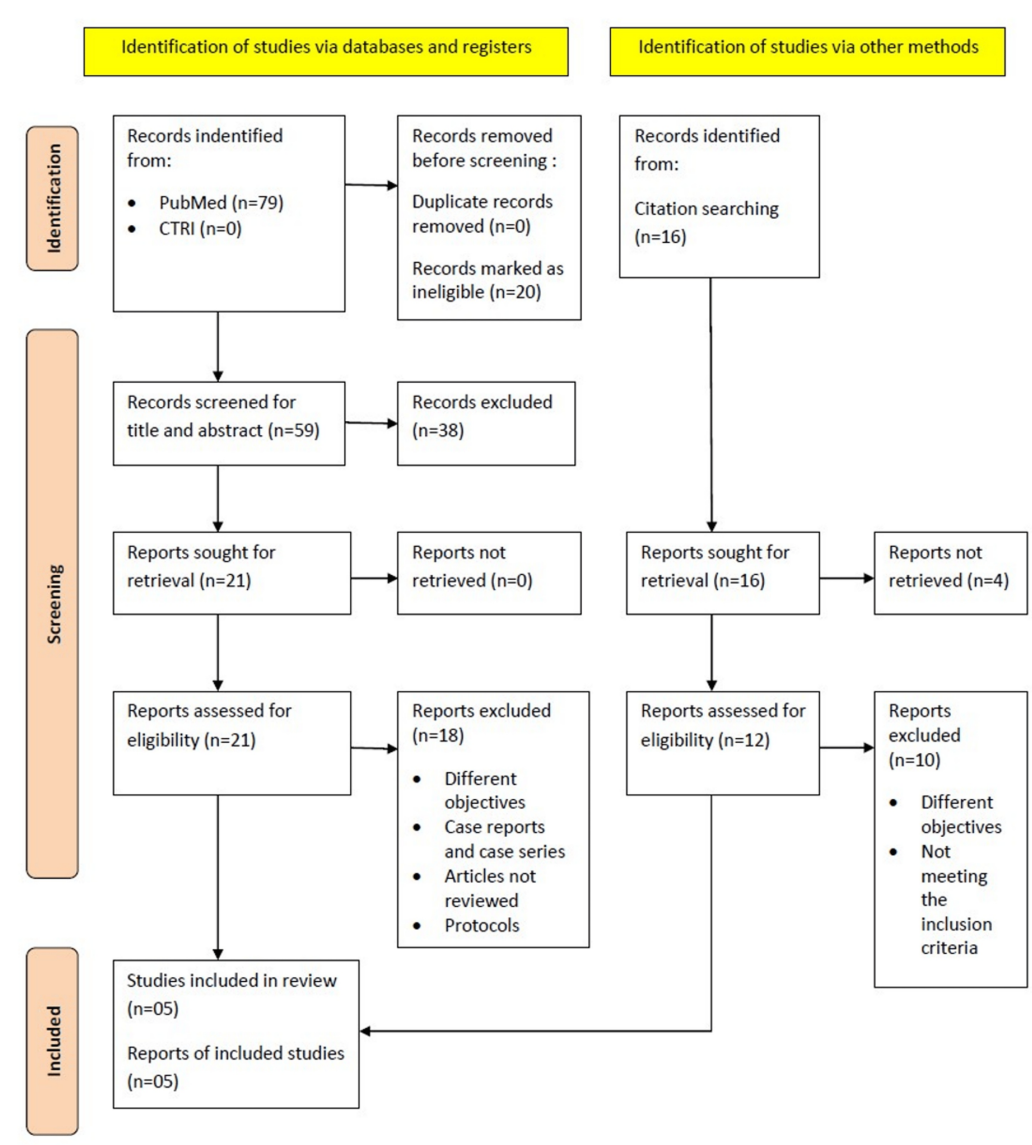
The PRISMA 2020 flowchart used in the systematic review (constructed by principal investigator) PRISMA: Preferred Reporting Items for Systematic Reviews and Meta-Analyses

We designed the research question as - whether supplemental high-flow oxygen (80% FiO_2_) during the perioperative period was effective in reducing the incidence of SSI following cesarean section. A pre-defined search strategy was used with the following combination of keywords: (((perioperative supplemental oxygen) OR (perioperative high flow oxygen)) AND (surgical site infection)) published in databases: Google Scholar, Scopus, Medline, PubMed, and Clinical Trial registry bodies since inception till April 2024. The full texts of all relevant manuscripts were retrieved. They were first checked for eligibility. Once found eligible, all articles were then checked by two authors independently. PRISMA guidelines were used to include the relevant studies for the review (Figure 1).

Methodology

For this systematic review, a search was made about the incidence of SSI after cesarean section and whether the administration of supplemental oxygen led to a decrease in the incidence of SSI. The variables searched were perioperative supplemental oxygen, high-flow oxygen, and SSI.

The initial search (((perioperative supplemental oxygen) OR (perioperative high flow oxygen)) AND (surgical site infection)) identified a total of 79 studies via searching through databases and clinical registries and 16 studies via manual searching of citations. Out of 79 articles, 20 were found to be ineligible for inclusion and 59 were screened for title and abstract. Out of them, 21 articles were sought for retrieval. After excluding unwanted articles, finally, five studies were included in the review.

Inclusion Criteria

Studies assessing supplemental (treatment arm) versus routine (control arm) FiO_2_ during cesarean section, scientific trials, and single- or multi-center randomized, or quasi-randomized controlled trials were included.

Exclusion Criteria

Study protocols without results, articles published in a language other than English, articles in pre-print, case reports, case series, case-control, cross-sectional and cohort studies, studies on the use of perioperative supplemental oxygen preventing SSIs in other surgeries like colorectal and transplant surgeries were excluded. The population-intervention comparison (control) outcomes and study (PICOS) design have been demonstrated in Table 1. 

**Table 1:** Tabular representation of the population-intervention-control-outcome-study (PICOS) type in the systematic review

PICOS	Criteria for inclusion in this systematic review
Population	Women undergoing cesarean delivery
Intervention	Women undergoing cesarean delivery with supplemental high-flow (80% FiO2) oxygen
Comparison	Patients undergoing cesarean delivery with 30% FiO2 (oxygen) or no supplemental oxygen therapy (control arm)
Outcome	Surgical Site Infection up to the first six weeks following cesarean delivery
Study designs included	Randomized Controlled Trials

Two authors (AS and NC) independently assessed all the articles that were found eligible for the study. The full texts of the articles were also analyzed. Any discrepancy with respect to study eligibility was discussed with the authors SPS and SP. Information was collected on variables related to the duration and route of perioperative oxygen administration, the flow rate of oxygen, the incidence of SSI in the study and control groups, and the total duration of follow-up. Quality assessment of all the recruited articles was done using Joanna Briggs Institute (JBI) Checklists and RevMan Plots. The relevant data were summarized and reported in the discussion section.

Results

Description of the Included Studies

All five included studies were randomized controlled trials. Among them, four studies were conducted in the United States (Ohio, Washington, Missouri, and Oregon) [5,8-10] and one in Panama [11]. The details of study objectives, study population, interventions, study outcome, and data assessment reporting are shown in Tables 2, 3. None of the studies were multicentric. In all five studies, supplemental oxygen used in the study arm was 80% FiO_2_. The sample size of the study ranged from 143 to 831 (Table 2). The duration of exposure to the supplemental oxygen was during and two hours after surgery in four studies and in one it was during and one hour after surgery. Gardella et al. verified the oxygen tension in the supplemental oxygen with venous blood gas analysis [8]. The cumulative incidence of SSI in the standard care group (control group) and supplemental oxygen group (study group) is tabulated (Table 3). Scifres et al. and Gardella et al. stated that the secondary outcomes of their study, such as hospital readmission, wound separation, and intravenous antibiotics, were similar in both groups [8,9]. Duggal et al. analyzed patients with diabetes separately and the rate of SSI in diabetic patients was 6.9% and 14.4% in the group who received 30% and 80% FiO_2_, respectively [5]. Gardella et al. performed a secondary analysis and found that hypertension, both chronic and gestational, was not a risk factor for peri-operative infection [8]. The secondary outcome stated by Admade et al. was the presence of respiratory complications prior to discharge from the hospital [11]. There were no respiratory complications in the groups. Scifres et al. sub-analyzed the patients’ compliance with oxygen masks at 60 and 90 minutes and found that there was no difference in both the groups [9]. Gardella et al. sub-analyzed the risk factors associated with infection and found that an increase in intra-operative blood loss was a leading cause of SSI in the early postoperative period [8]. In the same study, it was stated that other factors, like preterm labor, number of per vaginal examinations during labor, usage of any internal monitor, gestational hypertension or chronic hypertension, and tobacco usage in the antenatal period, were not associated with the risk factor of SSI. Williams et al. stated that three among their six covariates such as Caucasian race, increased body mass index and increased duration of surgery were associated with an increased risk of infection [10]. Scifres et al. studied the neonatal effects of additional oxygen administration during cesarean section [9]. They found that the umbilical artery pH and base excess were similar in both groups [9]. There were increased admissions in NICU in the supplemental oxygen group with eight and one neonatal death in the supplemental oxygen and control arm, respectively. However, none were attributed to oxygen supplementation. The duration of follow-up for diagnosing SSI in each study is also tabulated in Table 2.

**Table 2:** Results of the studies included in the review RCT: randomized controlled trial, L: liter, SSI: surgical site infection

S. no.	Study	Design	Sample size	Intervention	Duration of postoperative oxygen administration	Route of oxygen administration	Flow rate of Oxygen	Duration of follow-up	Inference
1.	Gardella et al. (2008) [8]	Single center RCT	143	80% vs 30% oxygen	For 2 hours	Non-rebreathing mask	15 L in both study and control groups	Daily during the hospital stay and on the 14th day	High-concentration oxygen did not decrease post-cesarean SSI
2.	Scifres et al. (2011) [9]	Single center RCT	585	80% vs 30% oxygen	For 2 hours	Nasal cannula and Non-rebreather mask	10 L in study group and 2 L in control group	2-4 weeks	Supplemental oxygen did not reduce post-cesarean morbidity, endometritis, and SSI
3.	Duggal et al. (2013) [5]	Single center RCT	831	80% vs 30% oxygen	1 hour	Aerosol face mask	10 L in both study and control groups	Daily during hospital stay, at 2 weeks and 6 weeks	No significant difference in SSI or endometritis in patients receiving 80% vs 30% oxygen
4.	Williams et al. (2013) [10]	Single center RCT	160	80% vs 30% oxygen	2 hours	Aerosol face mask	Not mentioned	Postoperative day 1,2 & 3, and 2weeks & 6 weeks postpartum	Perioperative high-flow oxygen was not effective in reducing post-cesarean SSI
5.	Admade et al. (2013) [11]	Single center RCT	326	80% vs no supplemental oxygen	2 hours	Oxygen mask with reservoir	Not mentioned	Every day during a hospital stay, day 15 and day 30 of post-cesarean	Supplemental oxygen did not reduce the incidence of SSI post-cesarean

**Table 3:** Percentage of occurrence of SSI SSI: Surgical Site Infection

Study	SSI in standard care group (control group)	SSI in supplemental oxygen group (study group)
Gardella et al. [8]	13.5%	19%
Scifres et al. [9]	8.8%	11.5%
Duggal et al. [5]	5.5%	5.8%
Williams et al. [10]	14.5%	13%
Admade et al. [11]	7.2%	5.4%

Methodological Quality of Studies

The authors assessed the quality of recruited studies with the aid of the JBI critical appraisal checklist [12]. The quality assessment of all the reviewed manuscripts (randomized controlled trials) was done separately. The risk of bias summary was assessed for each included study (as per the JBI checklist shown in Table 4) and the respective graphs were plotted using Rev-Man version 5.4 (The Cochrane Collaboration, London). 

**Table 4:** Quality assessment of included studies using the JBI critical appraisal checklist JBI: Joanna Briggs Institute

	Gardella et al. [8]	Scifres et al. [9]	Duggal et al. [5]	Williams et al. [10]	Admade et al. [11]
1. Was true randomization used for assignment of participants to treatment groups?	Yes	Yes	Yes	Yes	Yes
2. Was allocation to treatment groups concealed?	Yes	Yes	Yes	Yes	Yes
3. Were treatment groups similar at the baseline?	Yes	Yes	Yes	Yes	Yes
4. Were participants blind to treatment assignment?	Yes	Yes	Yes	Yes	Yes
5. Were those delivering treatment blind to treatment assignment?	Yes	Unclear	Yes	Yes	No
6. Were outcome assessors blind to treatment assignment?	Yes	Unclear	Yes	Yes	Yes
7. Were treatment groups treated identically other than the intervention of interest?	Yes	Yes	Yes	Yes	Yes
8. Was follow up complete and if not, were differences between groups in terms of their follow up adequately described and analyzed?	Yes	Yes	Yes	Yes	Yes
9. Were participants analyzed in the groups to which they were randomized?	Yes	Yes	Yes	Yes	Yes
10. Were outcomes measured in the same way for treatment groups?	Yes	Yes	Yes	Yes	Yes
11. Were outcomes measured in a reliable way?	Yes	Yes	Yes	Yes	Yes
12. Was appropriate statistical analysis used?	Yes	Yes	Yes	Yes	Yes
13. Was the trial design appropriate, and any deviations from the standard RCT design accounted for in the conduct and analysis of the trial?	Yes	Yes	Yes	Yes	Yes

During the quality assessment, all the randomized trials were found to have a low risk of selection bias as proper block randomization was used in the trial designs and the blinded sequences were uploaded in REDCap (Vanderbilt University, Nashville, TN) before the recruitment of participants. Although blinding of the gynecologist to the treatment assignment was not done by Admade et al. and was unclear in the trial conducted by Scifres et al., the risk of attrition, detection, and reporting bias was rated as low since the outcome data were complete and there was no selective reporting of the results.

Discussion

Globally, the incidence of SSI following cesarean section ranges from 0.7% to 23.5% [13-15]. Centers for Disease Control and Prevention defines SSI as infection in and around the skin where a surgical incision has been made. It can be classified as superficial SSI, deep incisional SSI, and organ space SSI. Since SSI is a computable outcome following surgeries, it has become a subject of concern for anesthetists and surgeons marking the commencement of “pay for performance” medicine. The burden of SSI has been estimated to increase the length of hospital stay by twofold thereby imposing an additional $16 billion per year on the US healthcare system [16]. The rate of admission to critical care units and mortality is almost twice as compared to the general population [17]. With the emergence of antibiotic resistance among nosocomial infections, prevention of SSI becomes the need of the hour.

Over the most recent years, SSI prevention has seized the spotlight of various quality enhancement projects [18]. SSI increases morbidity and mortality by approximately two times [17]. Oxidative killing by the neutrophils using molecular oxygen as its substrate acts as the chief defense against SSI [19], implying that tissue oxygen partial pressure over a clinical range provides resistance to infection. Supplemental oxygen also promotes angiogenesis [20], amplifies the levels of vascular endothelial growth factors [21], and aids in the conversion of fibroblasts into myofibroblasts, cells that mark the contraction stage of a wound recovery [22]. Increasing oxygen levels in a wound maximizes collagen deposition and its tensile strength [23]. The aforementioned mechanisms gave rise to the postulation that supplemental perioperative oxygen can decrease SSI frequency risk in contrast to lower routinely used inhaled oxygen concentrations during surgery and anesthesia. Since then, numerous randomized control trials have been conducted but unfortunately, we have been tangled in contradictory results from various randomized experiments.

Strengths

All the studies we found had used standard definitions of SSI and endometritis. Data collection was meticulous and organized. Randomization was properly done in all five studies. Subsequent data analysis used the intention-to-treat law in most of the included studies. Specifically, two well-conducted randomized trials by Duggal et al. and Scifres et al. were done on a large population size of 831 and 585 participants, respectively.

Limitations

All the studies reviewed had their own strengths and drawbacks. For instance, Scifres et al. did not measure circulating oxygen concentrations whereas Gardella et al. revealed that venous partial pressure of oxygen was greater in women who obtained a FiO_2_ of 80% as opposed to 30% through a non-rebreathing mask in females undergoing cesarean section. Nevertheless, both studies disproved any gain from supplemental oxygen in lessening the morbidity accompanying SSI, indicating that elevated circulating oxygen is perhaps not enough to avert infectious morbidity. Apart from this, one major limitation we could make was common to all these trials. They were all based on single-center data and no multicentric trials have been performed on this subject.

We also noticed some inequities in randomization structure with respect to presence as well as duration of labor and rupture of membranes. Since both of these factors have been independently correlated with an amplified risk for post-cesarean infectious morbidity [24-26], this negative aspect could not be overlooked even though Scifres et al. attempted to unveil that there was no deviation in the usefulness of supplemental oxygen in lessening the morbidity accompanying SSI centered on the presence of labor or rupture of membranes. Furthermore, a major technical limitation attributed to the various primary operating surgeons with a diversity of proficiency. This is awfully challenging but difficult to disregard.

## Conclusions

On the question of whether or not supplemental oxygen reduces post-cesarean SSI, all the studies we found did not show any considerable benefit of supplemental oxygen. One of the studies even analyzed the diabetic patients separately and found no difference in the two groups. We would like to conclude that the available literature is quite sufficient to prove that supplemental oxygen offers no added benefit in reducing post cesarean SSI and hence we do not recommend its use for this sole purpose. Instead, oxygen therapy during cesarean section should be centered exclusively on fetal and maternal physiological demands.
